# Microtube Array Membrane Hollow Fiber Assay (MTAM-HFA)—An Accurate and Rapid Potential Companion Diagnostic and Pharmacological Interrogation Solution for Cancer Immunotherapy (PD-1/PD-L1)

**DOI:** 10.3390/biom12040480

**Published:** 2022-03-22

**Authors:** Wan-Ting Huang, Tsao Yun, Chee-Ho Chew, Amanda Chen, Po-Li Wei, Kang-Yun Lee, Hsin-Lun Lee, Po-Hao Feng, Jeng-Fong Chiou, Ching-Mei Chen, Chien-Chung Chen

**Affiliations:** 1 Graduate Institute of Biomedical Materials and Tissue Engineering, College of Biomedical Engineering, Taipei Medical University, Taipei 110, Taiwan; sandyhuang@mtamtech.com (W.-T.H.); tsao1992@yahoo.com.tw (T.Y.); chchew88@gmail.com (C.-H.C.); 2 Department of Biochemistry, University of Washington, Seattle, WA 98195, USA; alc48@uw.edu; 3 Department of Surgery, Taipei Medical University Hospital, Xinyi District, Taipei 110, Taiwan; poliwei@tmu.edu.tw; 4 TMU Research Center of Cancer Translational Medicine, Taipei Medical University, Taipei 110, Taiwan; 5 The International Ph.D. Program for Cell Therapy and Regeneration Medicine, College of Medicine, Taipei Medical University, Taipei 110, Taiwan; leekangyun@tmu.edu.tw; 6 Division of Pulmonary Medicine, Department of Internal Medicine, Shuang Ho Hospital, Taipei Medical University, Taipei 235, Taiwan; fengpohao@tmu.edu.tw (P.-H.F.); 09135@s.tmu.edu.tw (C.-M.C.); 7 Division of Thoracic Medicine, School of Medicine, College of Medicine, Taipei Medical University, Taipei 110, Taiwan; 8 Department of Radiology, Taipei Medical University Hospital, Taipei 110, Taiwan; 132018@h.tmu.edu.tw (H.-L.L.); solomanc@tmu.edu.tw (J.-F.C.); 9 Graduate Institute of Clinical Medicine, Taipei Medical University, Taipei 110, Taiwan; 10 The Ph.D. Program for Translational Medicine, Taipei Medical University, Taipei 110, Taiwan; 11 The Ph.D. Program for Cancer Biology and Drug Discovery, Taipei Medical University, Taipei 110, Taiwan; 12 The Ph.D. Program in Drug Discovery and Development Industry, Taipei Medical University, Taipei 110, Taiwan; 13 The International Ph.D. Program in Biomedical Engineering, College of Biomedical Engineering, Taipei Medical University, Taipei 110, Taiwan

**Keywords:** PD-1/PD-L1+, immuno checkpoint blockers (ICBs), microtube array membrane-hollow fiber assay (MTAM-HFA), personalized medicine, companion diagnostic

## Abstract

Immunotherapy is one of the most promising forms of cancer treatment. In particular, immune checkpoint blockers (ICBs) represent some of the leading candidates which many drug developers have heavily invested in. During pre-clinical development and prior to human clinical trials, animal tests are a critical component for determining the safety and efficacy of newly developed ICBs for cancer treatment. In this study, we strive to demonstrate the feasibility of using hollow fiber assay microtube array membrane (MTAM-HFA) in the screening of anti-cancer ICBs. The MTAM-HFA process was carried out by encapsulating peripheral blood mononuclear cells (PBMCs) and the target cancer cells (cell lines or primary cells) and subcutaneously implanting them into Balb/C mice. At predetermined time points combination regimens of PD-1/PD-L1+ were administered accordingly and at a predetermined time point, the MTAMs were retrieved, and cell viability assays were carried out. The outcomes of the MTAM-HFA were compared against the clinical outcome of patients. Clinical comparison demonstrated excellent correlation between the screening outcome of MTAM-HFA of PD-1/PD-L1+ combination therapy and the clinical outcome of the lung cancer patients. Basic cell studies revealed that the utilization of MTAM-HFA in PD-1/PD-L1+ combination therapy revealed enhanced T-cell activity upon the administration of the PD-1/PD-L1 drug; thereby resulting in the reduction of tumor cell viability by up to 70%, and the cytotoxic effects by 82%. The outcome was echoed in the in vivo cell studies. This suggested that the MTAM-HFA system is suitable for use in PD-1/PD-L1+ screening and the accuracy, rapidity and cost effectiveness made it extremely suitable for application as a companion diagnostic system in both personalized medicine for cancer treatment and could potentially be applied to screen for candidate compounds in the development of next generation PD-1/PD-L1+ combination therapies.

## 1. Introduction

Recent advances in molecular science have revolutionized cancer treatment. Within the past decade, cancer treatment has shifted towards targeting of molecular targets that play a central role in the regulation of the immune response towards an active cancer. One such target is the PD1/PDL1 that has caught the attention of medical professionals worldwide and drug developers alike, with about 5000 related patents published on this novel cancer therapeutic to date [1]. This number is projected to continue to increase exponentially over the next few years as many drug developers have invested heavily in the development of novel PD1/PDL1 monotherapy or in combination with other drugs or immune targets [2]. To date, there have been up to 2000 clinical trials on anti-PD1 and 1000 clinical trials for anti-PDL1 [1]. In the year 2014, Nivolumab (Opdivo) and Pembrolizumab (Keytruda) was approved for anti-cancer therapy, and this approval was followed by cemiplimab (Libtayo), atezolizumab (Tecentriq), durvalumab (Imfinzi), and avelumab (Bavencio) by the US FDA [1,3]. 

Despite the hype surrounding the potential of anti-PD1/PDL1 therapies, not all patients respond favorably to these treatments. Apart from the poor response rate in certain patient segments, treatment-related adverse effects (TRAEs) and immune-related adverse effects (IRAEs) associated with neurological, respiratory, musculoskeletal, cardiac, ocular, cutaneous, gastrointestinal, hepatic, endocrine, and renal side-effects of anti-PD-1 therapy have been reported [1,4,5,6,7]. Notably, the adverse events (AE) of these treatments are mild when compared to the side effects associated with traditional chemotherapy. In order to improve the favorable response rate of patients receiving anti-PD1/PDL1 therapies, drug developers have begun to integrate small molecule targets into the traditional monotherapy treatment regimen of anti-PD1/PDL1, many of which are in the form of immune-modulators (nonpeptide small molecule inhibitors and peptide-based and peptidomimetic inhibitors) [8]. 

The limited success and disadvantages of antibodies prompted researchers to search for more effective strategies for PD-1/PD-L1 targeted therapy and improve the efficacy of cancer immunotherapy. Thus, studies on the discovery of low molecular weight compounds inhibiting the PD-1/PD-L1 interaction have begun to attract the attention of scientist. During the past 5 years, many companies, such as Arising International Inc., Chemocentryx Inc., Institute of Materia Medica, Guangzhou Maxinovel Pharmaceuticals Co., Incyte Corporation, Bristol Myers Squibb (BMS), and Aurigene, have discovered a series of small molecule chemical compounds and peptides. Meanwhile, these companies have applied for a series of patents related to inhibitors. Most of these patents presented not only the structure of PD-1/PD-L1 inhibitors, but also the method of compound synthesis and the use of inhibitors as immunomodulators. In addition, the patents showed verified inhibitory effects of these inhibitors. Some of these inhibitors could only block PD-L1/PD-1 interactions. Other inhibitors, such as the peptides discovered by BMS company (New York, NY, USA), could inhibit interactions of PD-L1 with PD-1 or CD80. All inhibitors discovered by Aurigene, including small molecule chemical compounds and peptides, showed an inhibitory effect on the PD-1 signaling pathway.

In view of such developments, and in combination with the high cost associated with anti-PD1 ICB therapy, there exists a dire need for predictive biomarkers/assays (especially in combination therapy settings) that can be utilized to accurately predict the potential outcome of such therapies. In a more traditional sense, the utilization of anti-PD1 under monotherapy settings entails the use of the FDA approved biomarker; expression of PD-L1 [9,10,11]. Despite such widespread use, certain studies have demonstrated the lack of correlation between the levels of PD-L1 expression and the clinical response of patients towards the administered ICB [12,13,14]. Even when patients exhibit negative PD-L1 levels, reports have indicated that clinical response rates range between 11% and 20%. Coupled with the inconsistency of assay systems and antibodies other factors include: the heterogenous and spatial nature of the expression of PD-L1 protein; differing positive cut-off values; and differing clinical response across different anatomy sites [15,16]. An example of differing response of ICB across multiple different anatomic sites can be seen in a study involving 398 metastatic NSCLCs where anatomic sites such as adrenal, lymph nodes and liver demonstrated the greatest response rates, while anatomic sites such as brain and bone demonstrated a lower response rate [9,17]. 

Additionally, other methods such as the tumor mutation burden (TMB)—which works on the basis of the potential for mutations in tumors to generate immune triggering neoantigens—have also been utilized to predict the response of cancer patients towards ICBs [18,19]. In these studies, it was demonstrated that the TMB method accurately predicted the response rate of cancer patients in cancer types with high tumor mutation burden (TMB-H), such as lung cancer, bladder cancer and melanoma with an objective response rate (ORR) of 39.8% (95% confidence interval; CI: 34.9–44.8) [18]. However, in other cancers such as breast cancer, glioma and prostate cancers where there is a lack of relationship between CD8 expression levels and cancer neoantigen. TMB-H did not accurately predict the ORR of patients suffering from these cancers [18]. It was concluded by the authors of these works that TMB levels did not accurately predict ORR in several types of cancers. 

Mismatch repair plays a vital role in identifying and repairing mismatched DNA bases. Genetic defects in the DNA mismatch repair protein often results in microsatellite instability which in turns leads to the accumulation of mutation load in the genes related to cancer; and ultimately, this results in the generation of neoantigens which stimulates the anti-cancer response of the host immune system [20,21]. Interestingly, high microsatellite instability (MSI-H) has been reported to be sensitive to ICBs, especially PD-1/PD-L1, with sensitivity of the tumor location and type being an independent factor that makes it an excellent predictor of response towards immunotherapies [20,22]. Despite these advantages associated with MSI, which seemed promising during the early stages of the utilization of MSI as a predictive biomarker in response to ICBs, several works have discovered that the benefit of MSI differs across different stages of cancers [23]. As an example, the ICB Nivolumab is suitable for the treatment of metastatic colorectal cancer at the advanced stage [23,24]. It was also concluded that despite the advances in MSI as a predictive biomarker in ICB response, much progress is still needed in the application of MSI to rare cancers, as well as the relationship between MSI, tumors and their classification [23,25]. 

The lack of a common consensus on the most suitable predictive system for anti-PD1/PDL1 combination therapy has led to a surge in demand for companion diagnostic systems capable of accurately predicting the clinical outcome of ICB therapies, especially in combination therapy settings [26,27,28]. This trend in development led us to adapt our previously demonstrated anti-cancer drug screening solution, that has been used to screen small molecule and protein chemotherapy drugs, for application in the screening of ICBs [29]. Briefly, the anticancer drug screening system outlined above is based on the novel microstructure known as a microtube array membrane (MTAM) which consists of one-to-one connected, ultra-thin hollow fibers arranged in an array formation. This platform has been applied in various applications ranging from encapsulated cell therapy, endotoxin removal, hemodialysis, fermentation, tissue regeneration and microbial fuel cells, in addition to the above-mentioned anticancer drug screening [29,30,31,32,33,34,35,36]. Hence, the purpose of this study is to demonstrate the feasibility of the MTAM-HFA system as a potential companion diagnostic for application in the screening of ICB combination therapies.

## 2. Materials and Methods

### 2.1. Electrospinning of the Polysufone (PSF) Microtube Array Membrane (MTAM) and Microstructural Characterization

The electrospinning process of the PSF MTAMs are as described in a previous work [29]. Two solutions which consisted of a core solution containing 10 wt% polyethylene glycol (PEG, Mw: 35,000; Sigma-Aldrich, Taipei, Taiwan) and polyethylene oxide (PEO, Mw: 900,000; Sigma-Aldrich) and; a shell solution which consisted of a 18 wt% polysulfone (PSF, Mw: 35,000; Sigma-Aldrich) dissolved in a co-solvent which consisted of tetrahydrofuran (THF; Sigma-Aldrich) and dimethylacetamide (DMAC; Sigma-Aldrich) at a ratio of 8:2 were prepared. The resulting solutions were co-axially electrospun with our internally designed spinneret at a voltage of 4.0–6.0 kV, a spinneret to collector height of 3.0–6.0 cm and a flow rate of between 4.0–10.0 mL/h (core solution) and 5.0–14.0 mL/h (shell solution) under ambient conditions. The resulting fibers were then soaked in double-distilled water (ddH_2_O) for 24 h, followed by a 12 h air drying process before being used in downstream characterizations/applications. The respective PSF MTAMs were sputter coated with gold and imaged with a scanning electron microcope (SEM; S-2400 Hitachi, Tokyo, Japan) at an accelerating voltage of 15 kV. The resulting SEM images were analyzed with Image J analytical software (Version: 1.8.0_172; NIH, MD, USA) to determine the basic microstructural parameters.

### 2.2. Cell Culture Preparation

The A549 cancer cell line (human lung adenocarcinoma) was expanded in RPMI-1640 medium (GIBCO, Gaithersburg, MD, USA) that was supplemented with 10% FBS (Biological Industries, Kibbutz Beit-Haemek, Israel). Antimicrobial agents amounting to 50 U/mL penicillin, and 50 mg/mL streptomycin (full medium, Biological Industries, Kibbutz Beit-Haemek, Israel) were added to the culture medium and maintained in an incubator at 37 °C in a 5% carbon dioxide atmosphere.

### 2.3. Isolation of Peripheral Mononuclear Cells (PBMCs) from Whole Blood

PBMCs were isolated as per the Ficoll protocol as outlined in [37]. Whole blood derived from healthy donors was maintained in an EDTA vacutainer (Sigma Aldrich, St. Louis, MO, USA), transferred and mixed with phosphate buffer saline (PBS, Sigma Aldrich) at a ratio of 1:1 in a separate centrifugal tube. Next, 3 mL of Ficoll-Paque solution (human: density:1.077 g/mL; mouse:1.083 g/mL. GE Healthcare, Taipei, Taiwan) was transferred to the bottom of another centrifuge tube. Carefully, 9 mL of whole blood-PBS solution was layered onto the surface of the Ficoll-Paque solution, and the entire layered solution was centrifuged at 400× *g* for 20 min. The PBMC layer was removed for downstream use. Next, 100–200 µL of cell suspension was transferred and mixed with an equal volume of Trypan Blue (Sigma Aldrich) and gently mixed via pipetting. A total of 5–10 µL of the mixture was transferred to a hemocytometer, and the number of viable cells were determined under a fluorescent microscope.

### 2.4. T Cell Activation and Flow Cytometry

The activation of the PBMCs involved the administration of 1 × 10^6^/100 µL A549 cancer cells via the tail vein into a standard Balb/c mouse. After 3 days, a 2 mL sample (per mouse) of whole blood was retrieved via cardiac puncture and the respective PBMCs were isolated according to the procedure outlined above. The resulting PBMCs were used either in downstream cellular studies or in flow cytometry to quantify the number of activated T-cells via the CD44 marker. For PBMCs that were designated for downstream use, the respective PBMCs were plated in standard 6 well plates (Biomann Scientific, Taipei, Taiwan) and maintained in RPMI-1640 medium (BD Bioscience, Qume Dr, SJ, USA) at 37 degrees Celsius and 5% atmosphere until ready for use, as outlined in the in vitro and in vivo section (within 30 min of isolation). As for the assessment of the activation of T-cells, flow cytometer was carried out based on the work of [38]. Briefly, PBMCs derived from different mice study groups (3 groups: Untreated Balb/C; Balb/C injected with A549 and Balb/C injected with A549 and anti-PD1/PDL1 (Bristol Myers Squibb, Taipei, Taiwan)) were fixed with 4% PFA/PBS for 30 min at 4 °C and immunostained with the corresponding antibodies (anti-CD44; BD Biosciences, Qume Dr, SJ, USA) and flow cytometer assessments of the % of activated T-cells (CD44 expression) were determined.

### 2.5. In Vitro and In Vivo Studies

Under in vitro or in vivo conditions, A549 cancer cell lines (ATCC, Manassas, VA, USA) and PBMCs (naïve/activated) were cultured in the respective settings: A549 cancer cell line (monoculture); A549 cancer cell line + naïve T-cells; A549 cancer cell line + activated T-cells; A549 cancer cell line + activated T-cells + anti-PD1 ICB drug. These were cultured either within the standard TCP or MTAMs as described previously in [29]. Briefly, for those cultured within the MTAM substrate, A549 (1 × 10^5^ cells/5 µL) and PBMC (2 × 10^5^ cells/5 µL) were siphoned into the lumens of the respective MTAMs. The respective edges of the MTAMs were folded over to create a seal on the top and bottom edges. The cell loaded MTAMs were swirled around in sterile RPMI 1640 medium (Sigma Aldrich) for 10 min to remove any excess cells that were left on the outer surface of the MTAM and subsequently transferred to a 10 cm TCP with RPMI 1640 culture medium (Sigma Aldrich). In the case of in vitro studies, the respective samples were cultured for up to 5 days at 37 degrees Celsius under 5% CO_2_ atmospheric conditions and at the predetermined time, samples were retrieved for cellular analysis to determine the viability and corresponding fluorescent imaging (CD45, E-cadherin and DAPI; BD Bioscience, SJ, USA). 

In the case of in vivo studies, the above-mentioned cell loaded MTAMs were subcutaneously implanted into the backs of balb/c mice. Skin incisions measuring 1–2 cm were made with a scalpel and the corresponding cell loaded MTAMs were implanted with the assistance of a microspatula After 24 h and with the absence of any negative presentation, the respective drugs at a concentration of 200 ng/20 g were injected via the tail vein as per conversion of human dosage to animal equivalent

### 2.6. Patient Primary Cancer Biopsy Samples and PBMC Collection

Primary biopsy samples of lung cancer patients between 20–80 years old were collected under IRB N201705050 and N201704053 for this study. Patients were diagnosed at the Department of Thoraic Medicine of Shuanghe Hospital, Ministry of Health and Welfare located in Zhonghe, Taipei, Taiwan. Biopsied surgical samples (<0.5 cm^3^) were immediately transferred into a sterile centrifugal tube containing RPMI medium and maintained at 4 degrees Celsius before being immediately processed for the downstream MTAM-HFA screening procedure. 20 mL of the respective patient’s whole blood was also collected for isolation of PBMCs as described above. 

### 2.7. The Microtube Array Membrane (MTAM)-Hollow Fiber Assay (HFA) Anticancer Drug Screening 

The procedure for the MTAM-HFA process was as described in [29]. Briefly, biopsied tumor samples were minced with a sterile scalpel. Collagenase/hyaluronidase (STEMCELL, cat#07912) enzymes were introduced into the culture medium at a ratio of 10:1 (culture medium: enzyme), and incubated at 37 degrees Celsius, under 5% CO_2_ atmosphere for 120 min. The digested tissue samples were then filtered with a sterile cell strainer (pore size: 70 µm; Fisherbrand, Waltham, MA, USA), followed by centrifugation at 300× *g* for 5 min before the number of viable cells were quantified (as described above). The resulting primary cancer cells and PBMCs were mixed in a ratio of 1:2. Next, the respective cell suspensions were siphoned into sterile PSF MTAMs measuring 0.5 cm × 1.5 cm at a concentration of 2 × 10^5^ cells/10 μL before having the respective ends folded over. The sealed membranes were then ‘swirled’ around in sterile PBS for 30 s before being incubated in RPMI culture medium at 37 °C in a 5% CO_2_ atmosphere. Within the shortest possible time, the respective membranes were subcutaneously implanted into the backs of standard Balb/C mice and treatment regimens were administered accordingly. At the end of the test cycle, the membranes were retrieved and the respective cancer cells and PBMCs were isolated via centrifugation at 670× *g* for 10 min. The separated A549 cancer cell lines and PBMCs were subjected to the MTT assay as described previously [29]. Angiogenesis assessment was carried out by quantifying the amount (%) of angiogenesis using image analysis software (Image J; NIH, Madison, WI, USA).

### 2.8. Response Evaluation Criteria in Solid Tumors (RECIST)

Response to the therapy regimens were converted to clinical outcome at the end of the respective treatment regimens and assessed based on RECIST in terms of the changes in tumor volume.

## 3. Results

### 3.1. In Vitro Comparison of the Mono-Cultured A549 Lung Cancer Cell Line and PBMCs in TCPs and MTAMs

**Figure 1:**
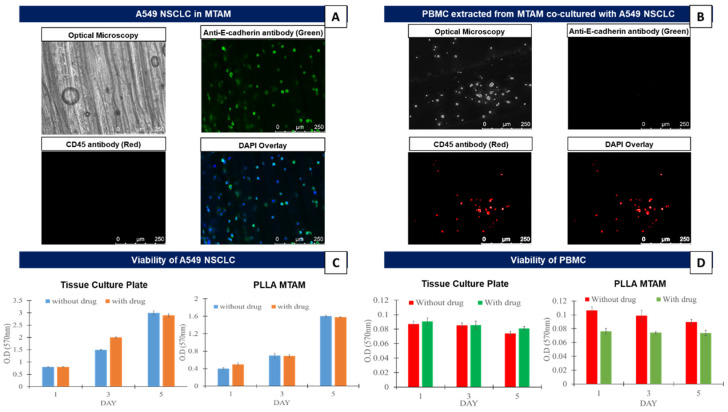
(**A**) Fluorescent microscopy of A549 cancer cell line co-cultured with PBMC in the PLLA MTAM; and extracted A549 cancer cell line stained with CD45 (non attaching cells), anti-E-cadherin (surface attaching cells) and DAPI. (**B**) Extracted PBMCs that were previously co-cultured with the A549 cancer cell line stained with the above outlined markers. (**C**,**D**) Comparison of the viability of the A549 cancer cell line and PBMCs that were monocultured in standard tissue culture plates (TCPs) and within the respective PSF MTAMs under two different conditions: with and without the corresponding anti-PD1 drug.

The results from this section revealed that when A549 (cell density: 1 × 10^5^) and PBMCs (cell density: 2 × 10^5^) were separately monocultured in the respective culture substrates, the cancer cells cultured within the MTAM substrate revealed a lower viability for both the cancer cells cultured with and without the presence of anti-PD1 drugs (Figure 1). However, it should be noted that the cellular proliferation of the cancer cells appeared to be consistently increasing without any loss of viability. Similarly, the monocultured PBMCs did not reveal any change in terms of viability throughout the entire 5 day observation period. The influence of the presence/absence of the anti-PD1 drug did not reveal any significant impact on the viability of the PBMCs cultured on the respective substrates. 

### 3.2. In Vitro Assay of the Co-Cultured A549 Cancer Cell Line and PBMCs Cultured in TCPs and MTAMs

**Figure 2:**
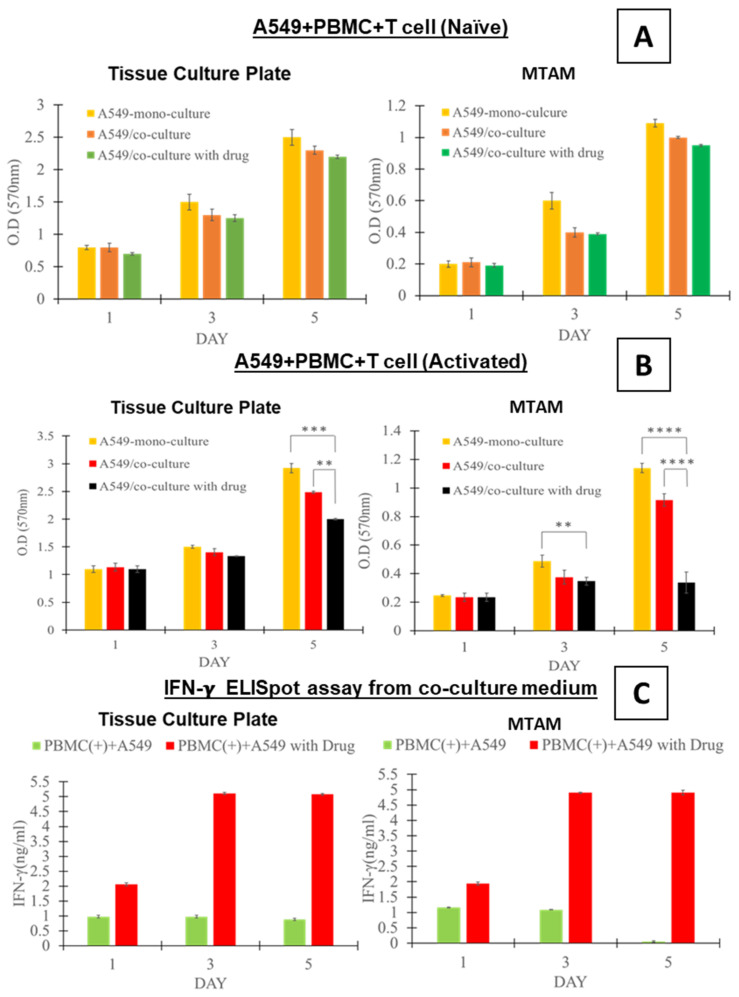
(**A**) In vitro comparison of A549 monoculture, A549 co-culture with PBMCs and A549 co-culture with PBMCs and anti-PD1 drug on day 1, 3 and 5 utilizing TCPs and PSF MTAMs. (**B**) Culture of A549 monoculture, A549 co-culture with PBMCs and A549 co-culture with PBMCs and anti-PD1 drug with the activation of T cells. (**C**) ElisaSpot assay of interferon gamma of the co-cultured cells in TCP and MTAMs. ** equivalent to *p*-value < 0.01; *** equivalent to *p*-value < 0.001; **** equivalent to *p*-value < 0.0001.

Generally speaking, the cancer cells cultured under monoculture and co-culture settings displayed a similar trend, with the cell viability of A549 increasing over the duration of the experiment (Figure 2A,B). At the respective time points, the viability of the A549 cancer cell line was greatest in the monoculture setting (Figure 2A,B), followed by those co-cultured with the PBMCs which contained naïve T-cells (Figure 2A) and activated T-cells (Figure 2B). The lowest viability of A549 cancer cells was for those co-cultured in the presence of anti-PD1. However, it should be noted that using statistical analysis, the difference in A549 viability revealed no significant difference (*p* > 0.5).

By the fifth day of the study, the A549 cancer cell lines that were co-cultured with the PBMCs (naïve T-cells) revealed a reduction of 8% (A549 + PBMC) and 12% (A549 + PBMC + drug) (Figure 2A). This trend was observed to be the same regardless of the culture substrate (TCP vs. MTAM) used in this study.

5 On the contrary, the for the A549 cancer cells cultured in the presence of PBMCs (activated T-cells; Figure 2B), the study group (cultured on TCP) without the presence of anti-PD1 revealed a reduction in viability by up to 14% and up to 19% (*p* < 0.01) when cultured with the presence of anti-PD1. Comparatively, when the similar groups were cultured within the MTAM substrate, the respective A549 cancer cell viability was significantly lower (19% for the group without drug and 70% for the group co-cultured in the presence of drug; *p* < 0.0001). Interestingly, for the cancer cells cultured in the above-mentioned settings, a significant difference was observable by day 3 between the A549 cancer cell lines cultured in a monoculture setting and those cultured in the presence of the anti-PD1 drug.

Interferon gamma levels, a key indicator of antitumor activity, revealed a significant difference between the A549 cancer cell lines co-cultured with PBMCs containing the naïve and activated T-cells (Figure 2C; *p* < 0.0001). A similar trend was observed between the TCP and MTAM culture plate with exception of the A549 cancer cell line co-cultured with naïve T-cells on day 5 using the MTAM culture substrate. 

### 3.3. In Vivo Assay of the Co-cultured A549 Cancer Cell Line and PBMCs Cultured within MTAMs

**Figure 3:**
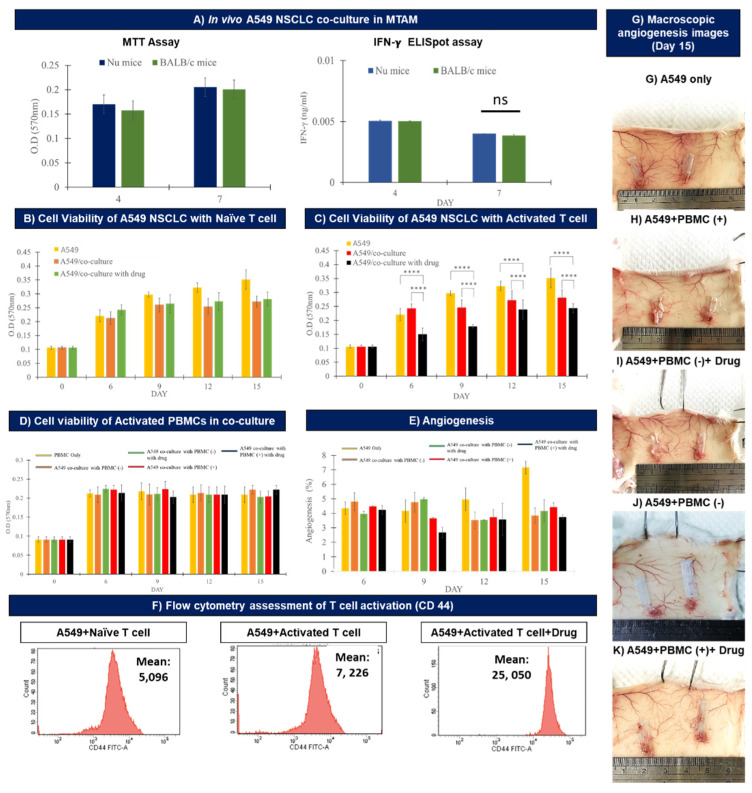
(**A**) Comparison of in vivo co-culture of the A549 cancer cell line within the MTAMs and its corresponding IFN gamma readings when utilizing the immune-compromised mouse and the standard Balb/C mice. No significant differences were found between the utilization of the two variants of mouse models. (**B**,**C**) In vivo study of the viability of the A549 cancer cell line + PBMCs with non-activated and activated T cells under various settings (monoculture, co-culture and co-culture with drug) (**** equivalent to *p*-value < 0.0001; ns: not significant). (**D**) Viability of the PBMCs in the monoculture and co-culture setting. (**E**) Angiogenesis degree levels of the subcutaneous blood vessel levels, which coincides with the macroscopic images of (**G**–**K**). (**F**) Flow cytometer analysis of the T-cell activation utilizing the CD 44 marker.

In order to determine the viability of the A549 cancer cell line when cultured within the MTAMs in different animal models we used nude mice and Balb/c mice (Figure 3A). By day 7, statistical analysis revealed no significant differences between the viability of the A549 cancer cell line implanted in either the nude mice or Balb/c mice. In terms of the interferon gamma levels secreted by the PBMCs, no significant differences were observed between the co-culture of A549 cancer cell line and PBMCs in MTAM that were subcutaneously implanted in nude mice or Balb/c mice. 

When A549 cancer cell lines were co-cultured with PBMCs (naïve T-cells; Figure 3B), the A549 cancer cell line registered the greatest viability across time among the three different culture settings (A549 monoculture, A549 + PBMCs (naïve) and A549 + PBMCs (naïve) + drug). It was also observed that the culture group containing A549 + PBMCs (naïve) registered a reduced viability of 19% (*p* < 0.01) when compared to the monoculture group. Interestingly, no significant (3%; *p* > 0.05) difference was found when comparing the viability of the A549 cancer cell line cultured with A549 + PBMCs (naïve) versus A549 + PBMCs (naïve) + drug. 

When co-cultured in the presence of PBMCs (activated T-cells; Figure 3C), a reduction of viability of the A549 cancer cell lines by up to 28.7% (*p* < 0.001) was detected. Conversely, in the presence of PBMCs (activated) + drug, the viability of the A549 cancer cell lines dropped by up to 40% (*p* < 0.001) when compared to the monoculture control. Critically, the viability of the PBMCs were not affected by the culture conditions (monoculture vs. co-culture), activation of T-cells, and/or the presence of the anti-PD1 immuno checkpoint blocker (Figure 3D).

In terms of angiogenesis (Figure 3E,G–K), on day 15 the greatest degree of angiogenesis was registered by the A549 cancer cell line monoculture group (Figure 3E). For the remaining study groups, the A549 cancer cell line cultured with the presence of PBMCs (activated T-cells) and in the presence of anti-PD1 immune checkpoint blockers registered the lowest degree of angiogenesis; with the remaining groups maintaining angiogenesis levels between 3–4%, where no significant differences between them were observed (*p* > 0.05).

Finally, for the flow cytometer assessment of the activation of the T-cells when co-cultured in the in the co-culture setting of A549 + PBMC (T-cell activation) + anti-PD1 drug (Figure 3F) assayed the expression intensity of CD44 marker as an indication of activation and cytotoxicity of the activated T-cells. In the absence of drug and when naïve T-cells were utilized, the mean reading registered a value of 5096, and this number registered an increase of 29.4% to a mean reading of 7226 when the naïve T-cells were replaced with activated T-cells. Lastly, in the presence of the anti-PD1 immune checkpoint blocker, the mean reading increased a further 71.2% to 25,050 when compared to the latter. 

### 3.4. Demonstration of MTAM-HFA (Anti-PD1) in the Clinical Setting

In the early stages of the clinical demonstration, primary biopsies derived from lung cancer patients were obtained and tested under three different culture settings in both in vitro and in vivo settings: monoculture; co-culture (lung cancer cells + PBMCs derived from the blood of the respective patient); and finally lung cancer cells + PBMCs + anti-PD1 immuno check point blocker. 

**Figure 4:**
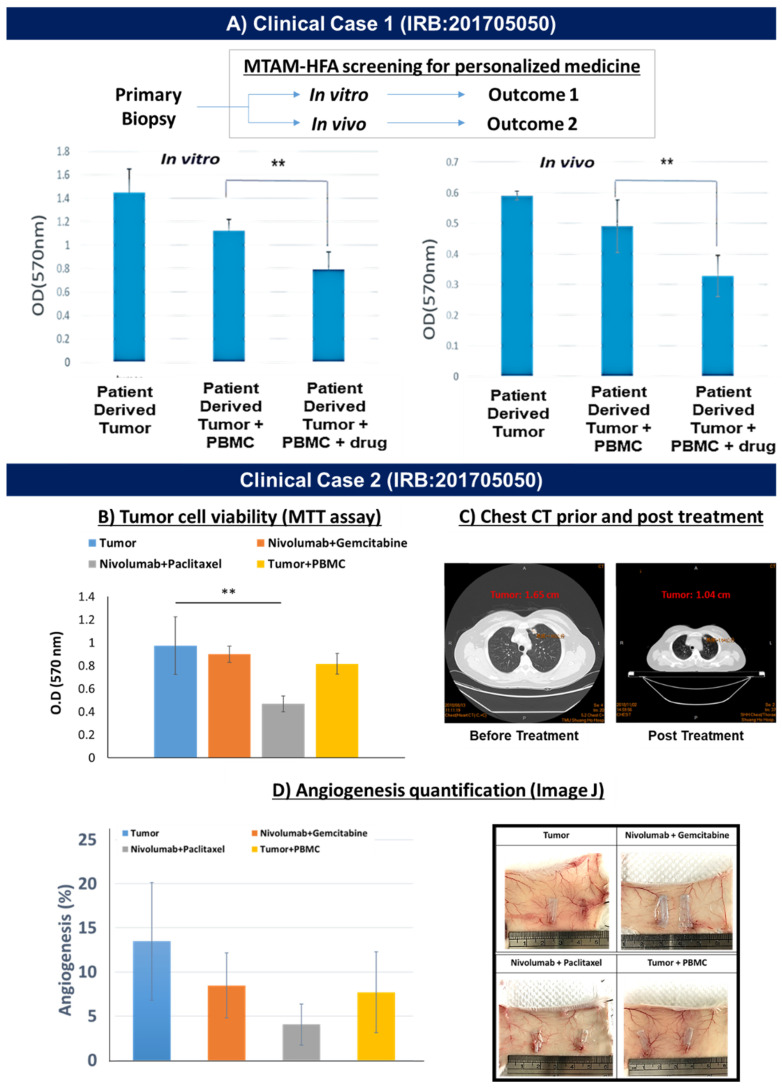
(**A**) Feasibility testing of the MTAM-HFA utilizing primary lung cancer cells which revealed a similar trend between the in vitro and in vivo groups. (**B**) Actual testing of the MTAM-HFA in personalized medicine for the personalized medicine application, successfully demonstrated correlation to the MTAM-HFA screening outcome (** equivalent to *p*-value < 0.01); (**C**) The resulting clinical outcome after the corresponding patient was administered the respective therapy which revealed a significant improvement in clinical outcome; (**D**) The angiogenesis assessment of the subcutaneous region of the balb/c mice utilized in the MTAM-HFA anti-cancer drug screening, which revealed a similar trend between the degree of angiogenesis and the viability of the primary lung cancer cells found in (**B**).

Under in vitro conditions, the cell viability of lung cancer cells were significantly higher than those cultured under the in vivo setting (Figure 4A). However, both exhibited similar trends in terms of the optical density (OD) of the lung cancer cell viability. Among the respective culture conditions (in vitro vs. in vivo), the reading of the monocultures registered the greatest reading of OD 1.4 (in vitro) vs. 0.6 (in vivo). Conversely, this reading registered a drop of 21% to OD 1.1 (in vitro) vs. 16% to OD 0.5 (in vivo). In the presence of the anti-PD1 drug, a further reduction of 28% (*p* < 0.001) in OD to a reading of 0.8 (in vitro) versus 30% (*p* < 0.001) reduction to OD of 0.35 (in vivo) was registered. 

Moving to an actual demonstration of the MTAM-HFA technology in personalized medicine for the screening of immune the checkpoint blocker (anti-PD1), we derived primary lung cancer cells from a recurrent lung cancer patient along with his/her whole blood sample for PBMC isolation. The resulting screening outcome revealed that the combination between Nivolumab + Paclitaxel revealed the greatest degree of inhibition of lung cancer cell viability when compared to the control (tumor only; Figure 4B). The recommended treatment combination was administered accordingly by the attending primary oncologist of the respective patient and after 3 months, the clinical assessment of the chest CT revealed a significant reduction of 37% in terms of tumor size from 1.65 cm to 1.04 cm (Figure 4C). The angiogenesis analysis (Figure 4D) of the subcutaneous region of the Balb/c mouse registered a similar trend which corresponded to those found in the viability assessment of the primary lung cancer tumor cells found in Figure 4B.

## 4. Discussion

In the initial phase, the A549 cancer cell line was monocultured within the MTAMs (Figure 1A). The resulting optical microscopy imaging revealed that the respective A549 cancer cell lines were observable, with the focus on the respective cells being gently adjusted along the *z*-axis to obtained a clear image, as described previously [29,30]. This confirmed the presence of the A549 cancer cell line that was successfully siphoned into the respective lumens of the MTAMs. When stained with E-cadherin, a surface attachment protein marker the A549 cancer cell lines that were attached to the unique inner lumen surfaces of the MTAM were clearly observed [39]. In combination with the viability assay of the A549 cancer cell line (Figure 1C), the viability of the A549 cancer cell lines registered an increasing trend, with no differences trend-wise between those cultured on TCPs or MTAMs. These findings coincided with our previous work which suggested that the ultra-short diffusion distance for nutrients, drugs and waste and the highly porous surface of the MTAMs, allowed signaling molecules to diffuse unhindered across the lumen wall. Additionally, the nature of the surface topography was highly beneficial for the adsorption of attachment proteins and subsequently the proliferation of the A549 cancer cells which made an excellent substrate for cell culture [40,41,42]. When co-cultured with PBMCs (Figure 1B,D), the culturing of PBMCs within the MTAMs did not significantly impact the viability of the PBMCs when by day 5, the viability was similar to those registered on day 1. This coincided with findings by another group which found that the PBMCs were generally viable for up to 21 days [43] and this duration was sufficiently long for our downstream MTAM-HFA which required a screening duration of 10–14 days [29]. 

When examining the viability of the A549 cancer cell line under monoculture settings (Figure 1C), the presence of the anti-PD1 drug did not impact the overall viability of the A549 cancer cell line as the absence of immune cells (T-cells) rendered the anti-PD1 drug useless in this setting [44,45]. Similarly, when cultured within the MTAMs, the viability of the A549 cancer cell line did not reveal any differences in viability even in the presence of the anti-PD1 drug from day 1 to day 5 (Figure 1D).

Under in vitro co-culture settings (naïve T-cells; Figure 2A) there were no significant differences in terms of the viability of the A549 cancer cell line when compared to the corresponding monoculture setting. A similar trend was observed for the co-culture of the A549 cancer cell line with naïve T-cells in the presence of the anti-PD1 drug. These findings could be attributed to the inactivated T-cells lacking the necessary recognition of the A549 cancer cell line and hence, the lack of an immune response in both the presence and absence of the anti-PD1 drug [46,47]. However, we did observe a relative difference in viability of the A549 cancer cell line when comparing the monoculture, A549 co-cultured with naïve T-cells and the A549 cancer cell line + naïve T-cell + anti-PD1 drug when comparing viability on day 1 versus day 5 (Figure 2A). We postulate that the co-culture of the A549 cancer cell line with inactivated T-cells will result in some degree of activation and result in a certain degree of cytotoxic response against the A549 cancer cell line, as seen in other works [48,49,50]. 

When the A549 cancer cell line was co-cultured with the activated form of T-cells (Figure 2B), the findings revealed a significant difference by day 5 in the viability of the A549 cancer cell line cultured in the monoculture setting versus those co-cultured with activated T-cell in the absence and presence of anti-PD1. We believe that the significant difference observed between the viability of the A549 cancer cell line cultured under a monoculture setting and those co-cultured with activated T-cells could be the result of T-cell recognition of the A549 cancer cell line that was conducted earlier, as described in the materials and method section. The in vivo activation of the T-cells most likely was the result of the introduction of the A549 cancer cell line into balb/c mice which is followed by the host immune response, where fragments of the A549 cancer cell line proteins were presented onto the surface of antigen presenting cells (APCs), and these in turn were utilized by immune memory forming B cells [51,52]. Consequently, when the PBMCs of the balb/c mouse were extracted for the in vitro assay in this section, the rapid recognition between the A549 cancer cell line and the activated T-cells will result in rapid cytotoxic activity of the T-cells. 

In the presence of the anti-PD1 drug, the viability of the A549 cancer cell line was the lowest among the 3 culture settings. It is well known that the anti-PD1 drug interferes with the immune stealth evasion by the A549 cancer cell line, where in the absence of anti-PD1 drug the APCs will bind to the antigens on the tumor cell and those of the T-cells. Consequently, this activates the T-cell receptor (TCR) and major histocompatibility complex (MHC) binding. The stroma of the tumor will in turn interact with the PD-1 present on the T-cells and this ultimately suppresses the T-cell mediated tumor cell cytotoxic effects [44,53].

The activation of the T-cells where the respective immune cells were exposed to the tumor antigens, as described earlier resulted in an adaptive immunity response. Hence, as described in other works the main source of interferon gamma in adaptive immunity is from the activation of T-cells, thereby describing the difference in interferon gamma levels between the naïve and activated T-cells, which accounted for up to five times difference (Figure 2C) [40,54]. This upregulation in the secretion of interferon gamma is crucial for cell mediated immunity, whereby the interferon gamma directly acts on a cytotoxic CD8 T-cell, and this in turn directly results in the induction of cytotoxic T-cell precursor proliferation and ultimately, the downstream cytotoxic effect on the A549 cancer cell line [55].

In terms of in vivo, we first established that there was no difference in terms of viability of the A549 cancer cell lines co-cultured with activated T-cells, and the corresponding interferon gamma levels secreted by the activated T-cells (Figure 3A). Such findings could be the result of the novel microstructure of MTAMs where the cancer cells are housed within the lumens of the MTAMs, as described in our previous work [29,41]. Through encapsulation within the lumens of our MTAMs, the host immune system would be prevented from having any form of physical contact between the host immune cells and the A549 cancer cell lines housed within. As most immune responses will require a physical contact between immune cells such as natural killer (NK) cells; dendritic cells and macrophages, this directly allowed us to utilize normal balb/c mice in our study. 

It should be noted that despite the lack of physical contact between the host immune cells and the A549 cancer cell lines housed within the lumens of the MTAMs, the ultra-porous surface of the lumen wall allows for the free diffusion of drugs and cytokine signaling molecules across the lumen membrane wall [32,42]. Additionally, when developing models for immunotherapy, a host with a functioning immune system is of utmost importance. The alternative solution would be genetically modified mice with humanized immune systems which are extremely expansive (50 times) the cost. Hence the MTAM based HFA system proved to be a highly cost effective and affordable model for application in anti-cancer drug screening. 

Under in vivo conditions, the culture of the A549 cancer cell line with naïve T-cells under monoculture, co-culture and co-culture with the presence of anti-PD1 drug (Figure 3B) revealed the same trend as those found in the earlier in vitro assays (Figure 2A). Similarly, when the naïve T-cells were replaced with activated T-cells (Figure 2C), the resulting viability of the A549 cancer cells revealed a similar trend as those found in the in vitro condition (Figure 2B) where the greatest viability of the A549 cancer cell lines were registered under the monoculture setting and the co-culture of the A549 cancer cell line with activated T-cell in the presence of anti-PD1 drug revealed the least viability. The mechanism of interaction describing these outcomes were similar to those discussed earlier in the in vitro setting. It was also observed that the viability of the A549 cancer cell line continued to increase from day 1 to day 15, and this trend was observed across all three different culture conditions, thereby suggesting that the anti-PD1 drug alone was not sufficient to inhibit the proliferation of cancer cells. 

It is also noteworthy to discuss that the viability of activated PBMCs (activated T-cells) remained throughout the entire experimental duration of 15 days. The stable trend in the viability of the activated PBMCs seemed to suggest that the MTAM substrate performed well based on the microstructural properties discussed earlier which were excellent for nutrient, waste, signaling and drug diffusion [41,42,45]. Additionally, the activated PBMCs within the lumens were derived from a separate host and if it were not for the presence of the MTAM, they would have been recognized as foreign by the immune system of the current host it was implanted in and eliminated. Therefore, the importance of the novel value proposition conferred by the MTAM cannot be over emphasized in this model system. 

In terms of angiogenesis (Figure 3E), the greatest readings were observed by the monocultured A549 cancer cell line. With the rest of the culture groups registering a significantly lower degree of angiogenesis. In the monoculture setting, the greatest viability of the A549 cancer cell line was observed (Figure 3C) and hence the corresponding secretion of vascular endothelial growth factor (VEGF) was the greatest; and this in turn was critical for the tumor proliferation, especially in the signaling of cancer stem cell initiation and proliferation [56]. When analyzing the macroscopic images of the implant site of the MTAM containing the A549 cancer cell line in various culture settings, the angiogenesis was clearly observable for all culture groups, as indicated in Figure 3E. Contrary to conventional techniques, often employed in patient derived xenograft (PDX) models, the analysis of angiogenesis when utilizing the MTAM-HFA system was very simple and straight forward, without any need for fixing and staining which significantly saves time, cost and reduces the possibility of sample destruction due to poor fixation and microtome cutting [57].

Flow cytometric assay of the in vivo sample of the activation of T-cells revealed that the greatest degree of activation was registered when the activated T-cells were co-cultured with the A549 cancer cell line and in the presence of anti-PD1 drug. The CD44 marker was selected as it is capable of distinguishing memory and effector T-cells that were activated from naïve conditions; with an additional role of binding to lymphocyte protein kinase which will further enhance the signaling of T-cell receptor [58,59]. The data from the flow cytometry were not surprising considering that the utilization of activated T-cells will result in a rapid immune–cancer response; and when in the presence of the anti-PD1 drug, the degree of activation was significantly greater and the inhibitory pathway of the A549 cancer cells were negated by the anti-PD1 drug, as described earlier in the in vitro section. 

In the first case in a clinical setting (Figure 4A), we derived primary biopsy samples of lung cancer patients for in vitro and in vivo assays. A side-by-side comparison revealed a similar trend when the primary biopsy of the lung cancer patient was screened for the response of the lung cancer patient. Based on this experience, we further performed an interventional case study on a recurrent lung cancer patient (Figure 4B). In this section primary biopsy samples were obtained and the attending oncologist gave us two possible combinations of anti-PD1 drug with a secondary chemotherapeutic cocktail. Within an extremely short timeframe of 10 days, we were able to determine that the drug Nivolumab (anti-PD1) and Paclitaxel were the most effective combination for this particular lung cancer patient. Based on this recommendation, the attending oncologist administered the recommended drug cocktail and 3 months later the resulting computer tomography (CT) scan of the lung cancer section revealed a significant reduction in terms of tumor size (Figure 4C), thereby suggesting that the screening outcome of the MTAM-HFA for immunotherapy combination treatment seemed to correlate with the clinical outcome of the patient. Additionally, the angiogenesis analysis (Figure 4D) that was conducted during the drug screening via the MTAM-HFA revealed a highly similar profile to those seen in the viability outcome of the MTAM-HFA (Figure 4B) 

## 5. Conclusions

The data derived from this study served as an early feasibility demonstration of the possibility of the use of the MTAM-HFA as a potential anti-cancer drug screening solution for anti-PD1/PDL1 + X combination therapies for lung cancer cases. With future clinical case studies, MTAM-HFA could potentially be a highly accurate, reliable, rapid and cost effective in vivo system that can be applied in both the personalized medicine and preclinical drug development setting. Undeniably, the clinical cases presented here were of case studies and hence, more through and complete trials should be performed in the future to fully establish the overall impact of this system in existing treatment protocols. 

## Data Availability

The data presented in this study are available on request from the corresponding author. The data are not publicly available due to privacy concerns and/or ongoing patent submission.
